# Evaluating portal-enabled elicitation of patients’ health-related values in solid tumor oncology: A qualitative content analysis

**DOI:** 10.1002/cncr.70159

**Published:** 2025-11-01

**Authors:** Carrie Sha, Erin Santos, Jaime Gilliland, Kathleen A. Lynch, William E. Rosa, Judith E. Nelson, Andrew S. Epstein

**Affiliations:** 1 Weill Cornell Medical College, New York, New York, USA; 2 Memorial Sloan Kettering Cancer Center, New York, New York, USA

**Keywords:** advance care planning, cancer, communication, goals of care, health information technology, patient portals, qualitative analysis

## Abstract

**Introduction:**

Electronic patient portals are increasingly used to improve care and patient–clinician communication. Portal-elicited health-related values (HRVs) were evaluated as to whether they contain themes similar to those arising from nurse-led in-person discussions.

**Methods:**

HRV questionnaires, consisting of seven questions, were sent by portal to patients of five solid tumor clinics from July 2023 to August 2024. Applying a theoretical framework generated from prior analysis of in-person nurse-led discussions with patients about their HRVs, questionnaires were coded via thematic content analysis.

**Results:**

A total of 1556 HRV questionnaires were sent to individual patients. Of 852 unique patients who returned questionnaires with response to at least one question, a random subset of 200 patients’ responses (167 gastrointestinal cancers, 33 genitourinary cancers) were analyzed by an interdisciplinary team. Analysis identified five themes consistent with those previously identified from in-person discussions: cancer as threat/disruption; expression of personhood and desire to retain a sense of self; communication with loved ones and the medical team as a tool for maintaining individual agency; connection to others as core to social identity and a source of individual strength; and sources of meaning and fulfillment. Uniquely identified by our analysis of portal responses were elements of financial toxicity and spiritual and existential well-being.

**Conclusion:**

Elicitation of patient values through the electronic portal provides valuable insights into the HRV of patients with solid tumors. The electronic portal captures values comparable to those elicited in in-person discussions, supporting efforts to scale such initiatives while preserving the content of patients’ reports as a basis for discussion of their HRVs with clinicians.

## INTRODUCTION

Understanding who patients are as individuals—beyond their diagnoses—is fundamental to high-quality oncologic care.^1–3^ Patients with solid tumors face complex medical decisions across the illness continuum, from diagnosis through treatment and, in some cases, end-of-life care. Yet, the clinical care environment often lacks structured mechanisms to surface what matters most to patients as they navigate cancer: their values, priorities, and sense of self. Our group has introduced the concept of a “values proposition” in cancer care, outlining the importance of integrating patients’ health-related values (HRVs) with oncologic decision making.^1^ We originally operationalized this approach through nurse-led in-person interventions, which included a structured method to elicit patients’ HRVs in outpatient oncology settings.^4–6^ Findings demonstrated that values elicitation is both acceptable and meaningful to patients, reinforcing the critical role of structured values communication to help patients feel heard and to ensure the high quality of clinical communication.

While in-person values discussions offer depth and personalization, they are resource intensive and may be difficult to scale across diverse oncology settings. Increasingly, electronic health tools, such as patient portals, are being leveraged to enhance care delivery, foster patient engagement, and support person-centered communication.^7–10^ Our team’s work has shown how structured communication through the portal can improve the quality and efficiency of cancer care, particularly in assessment of patients’ understanding of their illness and its treatment intent,^11^ and more recently, in the assessment of their HRVs.^12^ These studies suggest that patient engagement by portal may be a viable pathway for elicitation of HRVs to advance person-centered oncologic care. However, the potential for portals to replicate the relational and communicative depth of in-person encounters—particularly around HRVs—remains understudied.

In this qualitative study, we evaluated responses of patients with solid tumors to questions sent via electronic patient portal to elicit HRVs. We hypothesized that the essential elements of nurse-led in-person HRV discussions could be maintained in a portal-enabled format that preserves the integrity of person-centered elements of HRV responses while improving clinical efficiency. To test this hypothesis, we examined how patients responded to values-based questions delivered through the portal, analyzing what information they shared about themselves, and how themes from the portal communication compared with those from nurse-led in-person discussions about HRVs.^6^

## MATERIALS AND METHODS

### Study design

This was a qualitative study conducted as a preplanned component of an institutional quality improvement initiative, which the Memorial Sloan Kettering (MSK) Institutional Review Board reviewed and exempted from informed consent.

### Patient population

Between July 2023 and August 2024, 1556 questionnaires, each consisting of seven questions about health-related values (patients’ personhood, sources of strength, concerns, definition of living well, hopes, critical abilities, and if values have been discussed with a loved one) were sent through the MSK patient portal (*MyMSK*) to individual patients of five oncology clinics: four gastrointestinal (GI) and one genitourinary (GU).^12^ The HRV questions were similar to those asked of patients in person in our prior research in medical oncology^4–6,13^ (identical with the exception of a question about what patients would want in an “unexpected crisis situation,” which our research team removed precautionarily when values elicitation moved from nurse-led in-person discussions toward portal-elicited methodology) and similar to those in preoperative^14,15^ clinical settings at MSK. This automated process of sending HRV questions facilitated implementation, adoption, and sustainability of questionnaire delivery^16^ and was deployed in response to patient and clinician feedback we previously described.^10^ Of the 852 unique patients (the majority of whom had GI cancer) who returned questionnaires with response to at least one of the questions, a random subset of 200 responses (a number sufficient for saturation, accounting for variability in patient demographics^17–19^) was analyzed for the present study. Although this was random and not intentional sampling, diversity of representation based on demographic and clinical factors was present (Table 1). Patient respondents were assigned a study identification number and randomly selected for analysis using a random number generator. Patient characteristics were reported using descriptive statistics.

### Qualitative analyses

Responses provided from patients via portal were analyzed using thematic content analysis.^20,21^ Such a qualitative descriptive design is appropriate given our prior conceptual framework now being examined through the lens of portal-enabled HRV elicitation, described in detail later.^22,23^ We assembled an interdisciplinary analysis team, led by a qualitative methods specialist (J.G.) who provided training on coding procedures to three additional study team members: a GI medical oncologist (A.S.E.), a surgical resident physician (E.P.S.), and an internal medicine resident physician (C.S.). A nurse behavioral scientist (W.E.R.) supported the analysis team in synthesizing thematic findings.

This analysis consisted of three phases. First, we developed an a priori codebook derived from our previously developed theoretical framework of patients’ navigation of a cancer diagnosis.^6^ This framework was generated through a grounded theory analysis of in-person HRV discussions with oncology office practice nurses and consists of five thematic constructs: (1) the existential disruption of cancer frames patients’ expression of their values, (2) expression of character (personhood) and a desire to retain a sense of self, (3) communication with loved ones and the medical team as a tool for maintaining individual agency, (4) connection to others represents a core social identity and source of individual strength for patients, and (5) sources of meaning and fulfillment arising in the face of the cancer diagnosis.^6^ Guided by this framework and facilitated by a data matrix, team members independently coded a subset of five unique patient responses to ensure consistency in code application. Discrepancies in code assignment were resolved via consensus discussion. Then, each coder was assigned 50 additional patient responses to code independently. After all data were coded, two analysts (J.G., E.P.S.) performed quality assurance checks on all coded data, resolving discrepancies and identifying emergent areas of interest not included in the a priori codebook. In the final phase, the analysis team collaboratively reviewed each code, identifying key content, categorizing codes, and identifying congruence and divergence of themes observed across portal responses compared to those identified in nurse-led discussions.^6^

## RESULTS

Analysis included 167 patients with GI cancers and 33 patients with GU cancers. Most patients identified as non-Hispanic White, with either colon, pancreas, or GU cancers. The median age of patients was 67 years and 41% had metastatic disease (Table 1).

Qualitative analysis identified five themes consistent with those previously identified from in-person discussions^6^: (1) cancer as existential threat/disruption which frames patients’ values, (2) expression of personhood and desire to retain a sense of self, (3) communication with loved ones and the medical team as a tool for maintaining individual agency, (4) connection to others as core to social identity and a source of individual strength, and (5) sources of meaning and fulfillment. Uniquely identified by the present analysis were elements of financial toxicity (embedded in theme 1) and spiritual as a source of resilience (embedded in theme 5). Next, we characterize these themes within the context of portal-elicited responses.

### Theme 1: Cancer as threat/disruption – The existential disruption of cancer frames patients’ expression of their values

Patients’ responses to HRV questions depicted the experience of cancer as a significant existential disruption, forcing them to confront what matters most. Uncertainty about how their disease may progress or recur, fears about how treatment may affect their physical and emotional well-being, and fears about treatment not working provided a lens through which to frame the expression of HRVs. Bodily autonomy was a paramount concern, such as changes to cognitive functioning or the ability to care for oneself, as were worries about maintaining a sense of normalcy through routine activities like work or time with family. A 72-year-old woman with colon cancer reported the critical physical abilities she could not live without, “Self-care… intact mental status. The ability to use my brain to make decisions…learning abilities and skills to be/remain active in professional activities… [have] social interaction. The ability to enjoy family interaction.” A 35-year-old man with stomach cancer described that living well for him included being pain free and stated his desire “to do things without pain, and still maintain a healthy diet and enjoyment of my interests and hobbies.”

Echoing concerns related to dying highlighted in the nurse-led values discussions,^6^ patients conceptualized possible death from cancer as a disruption to futures they had planned. A 75-year-old man with pancreatic cancer shared a desire to “not leave things unfulfilled,” whereas a 56-year-old man with colorectal cancer stated that dying from cancer would cause him to leave his family “prematurely.” This informed worries about transferring familial roles onto other members of the family, such as financial responsibilities. A 67-year-old man with bladder cancer stated being concerned about dying and “how will it affect my family. We have a son who’s autistic and living a semi-independent life in [another state]. He will always need some assistance. It’s a fabulous place but very expensive. […] We’ve set aside a trust fund [for our daughter] so she’ll be able to take care of [him].”

### Theme 2: Character – Expression of character (personhood) and a desire to retain a sense of self

Patients highlighted the importance of functional independence as a predominant aspect of identity to be maintained. This was expressed across HRV questions, from those about personhood to concerns and hopes to critical abilities. Independence was often connected to a desire to avoid being a burden to others while navigating cancer, with an 80-year-old man with intrahepatic bile duct cancer reporting that his cancer care “is a huge imposition on my wife. Caring for me is time-consuming, disruptive to both our lives, and family.”

Patients also framed their health care preferences and goals within the context of their identity, demonstrating how identity is often inextricably linked to values and preferences such as for communication or approaches to cancer diagnosis and treatment. A 77-year-old man with small intestine cancer reported that he was “easy going and [a] good listener. I like to hear an honest assessment and how to approach treatment.” A 67-year-old man with pancreatic cancer stated, “I want to remain as independent as possible. I am interested in my condition, so I would like it explained as simply as possible.”

### Theme 3: Communication with loved ones and the medical team as a tool for maintaining individual agency

Communicating values and preferences with family and loved ones allowed participants to uphold their autonomy, such as at the end of life, with a 56-year-old woman with a metastatic high-grade well-differentiated neuroendocrine tumor stating, “I don’t want a mass. I want friends and family members to bring pictures and reminisce. Good food and wine/beer. I don’t want anyone sad.” A 72-year-old woman with metastatic carcinoid tumor described the importance of these discussions with regard to patients’ autonomy, stating, “my husband and I have had numerous discussions about what I want/need in life and the choices that I have made for later in life (and death).”

Participants also leveraged the patient portal to inform the care team of their communication preferences to guide the content and depth of information discussed. Some patients desired comprehensive data, whereas others were ambivalent about receiving detailed statistics about the diagnosis and treatments. A 37-year-old woman with intrahepatic bile duct cancer reported, “I am a doctor and like to know all of the data when making decisions,” whereas another patient, a 75-year-old woman with small intestine cancer, stated that “I want to remain optimistic” but learning about prognosis-related statistics may induce fear and “could be inappropriate for my immune system and general positive outlook.” Sharing and having these preferences respected often restored a sense of control for patients in an otherwise vulnerable situation. When asked what gives her strength in facing cancer, a 44-year-old woman with colorectal cancer shared, “I realize my [diagnosis] is out of my control, but I can control how I react to it all and gather all the information I can.”

### Theme 4: Connection to others represents a core social identity and source of individual strength for patients

The idea of connection was central to patients’ responses, both to loved ones and to their medical team. Patients described drawing significant support from family and friends while navigating their cancer diagnosis, often connecting family to the idea of “living well.” A 57-year-old woman with pancreatic cancer described the importance of “being able to laugh and love my daughter and husband the way we always did before cancer.” In fact, for many patients, a connection to loved ones was cited as something they felt they could not live without. A 56-year-old woman with pancreatic cancer described, “I can’t imagine living without […] spending quality time with my family,” whereas a 72-year-old man with pancreatic cancer shared the importance of “being able to hold my wife.” Patients cited desires to participate in family milestones, such as seeing children grow up, or witnessing graduations, weddings, and births. A 41-year-old man with intrahepatic bile duct cancer shared his hope to witness his “daughter’s basketball go to the next level.”

Connection also extended to the medical team, with patients sharing that this connection is both an important part of their identity and a source of strength. Patients often took the opportunity to share the importance of the connection to their medical team, such as desires to be treated with dignity and “to be recognized as a person and not a number and any concerns be dealt with honestly” (76-year-old woman; bladder cancer). Patients also described the trust they have in their medical team, allowing them to “hand over control” (74-year-old woman; intrahepatic bile duct cancer) of a difficult situation. A 78-year-old woman with pancreatic cancer shared, “I find strength in the counsel and encouragement from the caring and kind medical professionals I am fortunate to have treating me.” Others described faith in the medical institution and how this gives them great confidence as they face cancer.

### Theme 5: Sources of fulfillment and life’s meaning

Patients incorporated previously identified constructs of character, connection, and communication into their experiences of finding meaning and fulfillment while navigating a cancer diagnosis. Patients most often conceptualized “living well” as the ability to maintain a sense of normalcy. For some patients this meant continuing to work, whereas others focused on staying healthy and feeling physically well. Many described a desire to be independent and perform activities of daily living, such as one 68-year-old man with pancreatic cancer who described normalcy as the “ability to eat, sleep, move about and socialize.” Responses highlighted the importance of finding fulfillment and purpose in everyday life while facing cancer, such as enjoying the present moment through hobbies or spending time with loved ones. Patients often shared their desires to travel, engage in physical activities, and raise their children or spend time with grandchildren. A 67-year-old man with small intestine cancer described what living well means to him, sharing, “feeling well when I wake up every morning, to start, and feeling well throughout the day, and living modestly and comfortably, without physical and mental anchors to weigh me down. Vacation once a year, walks with my wife and dog.”

Patients also cited spirituality or mental fortitude as sources of resilience central to their experiences of finding meaning and fulfillment amid the challenge of navigating a cancer diagnosis. Some defined their personhood through their spirituality and described leaning on their faith or faith communities as sources of strength, desiring to maintain spiritual practices such as meditation or drawing on prayer as a source of peace. A 75-year-old woman with small intestine cancer shared, “my faith has been leaned on more than ever, and much strength has come from that.” Others characterized themselves as optimistic, making a concerted effort to stay positive or hopeful. A 64-year-old man with bladder cancer was emboldened to share, “I am stronger, tougher and more optimistic than any patient you’ve had before.”

## DISCUSSION

Our qualitative descriptive analysis demonstrated that portal-elicited HRV themes are similar to those identified in our prior study focused on nurse-led HRV discussions.^6^ These findings advance our previous work that originally illustrated the effectiveness of portal questionnaires in eliciting understanding of illness and treatment goals in patients with GI cancer without causing patient distress.^11^ Building on this foundation, we demonstrated that the portal platform can allow patients to effectively convey their thoughts and feelings as it relates to what matters most to them. Patients responding to portal questions did so in detail and usually answered most of the questions sent to them,^12^ suggesting an ease and comfort sharing their values with their team over an electronic interface. This is also consistent with our prior pilot analyses demonstrating all patients reporting comfort with communicating their HRV over the portal,^11^ and the lack of patient distress reported to physicians and nurses, who viewed all patients’ responses and addressed them in clinic.^12^

We identified two unique aspects of portal responses not seen in prior in-person discussions: financial concerns and both spiritual and existential well-being. Financial concerns were reflected in patients’ concerns regarding the financial well-being of family members both before and after their death. Additionally, although our patient population was mostly not from underserved communities, financial toxicity is a common and debilitating concern for patients with cancer and disproportionately affects disadvantaged populations.^24^ Given the stigma of financial toxicity, patients may be hesitant in discussing these difficulties.^25,26^ The portal, as opposed to a face-to-face encounter, may allow patients to feel more comfortable in revealing financial concerns.

We gleaned both spiritual and existential well-being as core sources of meaning for patients, who expressed the importance of their faith, faith communities, sustained spiritual practices, and the state of overall optimism, peace, and contentment. Spirituality is an important aspect for patients coping with serious illness and incorporating spiritual care into patient-centered practice has been shown to improve health outcomes.^27^ Existential well-being is also a key element of personhood that is often overlooked by providers and is brought to the forefront when patients cope with life-threatening illnesses.^28,29^ A better understanding of patients’ existential needs improves quality of life^30^ and is central to person-centered oncologic care. Like financial concerns, patients may have been more inclined to share these highly personal details on a portal platform, without the pressure of face-to-face interactions including anticipating the immediate reactions of practitioners, and with the added benefit of more time to deeply consider their responses when responding over the portal.

These examples suggest that the portal method for eliciting HRV not only yields comparable responses from patients but also provides potential advantages as a platform for values communication. Portal-based elicitation of HRV may have allowed patients to be more open discussing sensitive concerns and afforded them more reflective time to thoroughly consider what matters most. Unlike prior in-person discussions with office practice oncology nurses who summarized patient responses into the electronic health record (after affirming summaries with patients^4,5^), the portal also empowered patients to use their own words in describing their true thoughts and feelings.

As such, portal-elicited HRV methodology has many implications for real-world integration into the clinic. Participating clinicians receive a copy of patient responses before their visit and can use it to adjust their communication styles, management decisions, and set goals for the visit. Portal elicitation of HRVs can save significant time in clinician visits while providing invaluable guidance for clinical teams to meet patient priorities. Because of the ease and widespread use of the patient portal,^8^ this method of eliciting HRV can be scaled to include more oncology clinics in various practice settings. Ongoing implementation studies will address pertinent unanswered questions such as how clinical teams use portal responses in discussing and documenting patient values, and how patients feel about their HRVs being elicited via portal compared to in-person discussions. Additionally, this study provides a foundation for future analyses of larger datasets of portal-elicited HRV, including associations of HRVs with different patient demographics and levels of prognostic awareness. This is nonetheless the first qualitative analysis of portal-elicited HRV in patients with solid malignancies after an initial diagnosis. Our findings demonstrate the richness of portal-elicited HRVs and show how clinicians can use the electronic portal as a tool in providing person-centered oncologic care.^31,32^ Other strengths include the use of a rigorous qualitative methodology, the diverse skillsets and perspectives of a highly multidisciplinary team, our robust sample size of 200 unique patient responses, and the variety of cancer types included in our analysis.

Our study has limitations. We analyzed a randomly selected patient subset and did not stratify or formally address possible values differences within all demographics (e.g., age, stage). This will be the focus of a future mixed methods study of this entire 852 patient respondent population. Additionally, each patient’s ability to respond to the portal questionnaire depends on familiarity with and accessibility of the internet, English comprehension, and health literacy. Thus, as reflected in our predominantly non-Hispanic White patient sample, our study design may favor those who are primarily English language-speaking and health literate, missing vulnerable patient populations. Furthermore, our study was conducted at a single dedicated cancer center at five oncology clinics, which may limit transferability of findings to other contexts. To address these disparities, we are currently studying the implementation of a Spanish HRV questionnaire for Spanish-speaking Latino patients^33^ and planning in-depth implementation and effectiveness testing in a multisite investigation. Although we have previously demonstrated that all participating clinicians viewed patient responses and deemed them valuable,^12^ further study will benefit from gathering detailed clinician feedback on how portal responses affect workflow and the provision of patient-centered oncologic care.

## CONCLUSION

Elicitation of patient values through the electronic portal provided valuable insights to HRV of patients with solid tumor diagnoses. Our study demonstrates the feasibility and benefits of eliciting HRV through a portal-elicited process, including the identification of two unique aspects of portal responses not seen in prior in-person discussions: financial toxicity and both spiritual and existential well-being. Our findings suggest that the electronic portal is an effective method for eliciting HRVs, supporting efforts to scale such initiatives while preserving the content of patients’ reports as a basis for discussion of their HRVs with clinicians. Ongoing research investigates the optimal clinical implementation of portal-enabled HRV elicitation and effects on patient-centered oncologic care.

## Figures and Tables

**TABLE 1:** Patient characteristics.

	(*N* = 200)
Age at questionnaire, years – median (range)	67 (28–87)
Sex	
Male	104 (52%)
Female	96 (48%)
Race	
White	157 (79%)
Black	21 (11%)
Asian	11 (5%)
Other	4 (2%)
Not reported	7 (3%)
Ethnicity	
Non-Hispanic	173 (87%)
Hispanic	11 (5%)
Not reported	16 (8%)
Marital status	
Divorced	18 (9%)
Life/domestic partner	3 (2%)
Married	133 (66%)
Separated	3 (2%)
Unmarried	27 (14%)
Widowed	16 (7%)
Religion	
Buddhist	2 (1%)
Catholic	96 (48%)
Other Christian	41 (20%)
Jewish	24 (12%)
Muslim	1 (1%)
None	30 (15%)
Other	2 (1%)
Unknown	4 (2%)
Cancer type	
Colon/anorectal/intestinal	92 (46%)
Pancreatic	45 (23%)
Genitourinary	33 (17%)
Hepatobiliary	27 (13%)
Gastroesophageal	3 (1%)
Cancer stage	
4	82 (41%)
3	63 (32%)
2	28 (14%)
1	24 (12%)
0	3 (1%)

## Data Availability

The data that support the findings of this study are available from the corresponding author upon reasonable request.
